# Tuning characteristics of low-frequency EEG to positions and velocities in visuomotor and oculomotor tracking tasks

**DOI:** 10.1038/s41598-018-36326-y

**Published:** 2018-12-07

**Authors:** Reinmar J. Kobler, Andreea I. Sburlea, Gernot R. Müller-Putz

**Affiliations:** Institute of Neural Engineering, Graz University of Technology, Graz, Austria

## Abstract

Movement decoders exploit the tuning of neural activity to various movement parameters with the ultimate goal of controlling end-effector action. Invasive approaches, typically relying on spiking activity, have demonstrated feasibility. Results of recent functional neuroimaging studies suggest that information about movement parameters is even accessible non-invasively in the form of low-frequency brain signals. However, their spatiotemporal tuning characteristics to single movement parameters are still unclear. Here, we extend the current understanding of low-frequency electroencephalography (EEG) tuning to position and velocity signals. We recorded EEG from 15 healthy participants while they performed visuomotor and oculomotor pursuit tracking tasks. Linear decoders, fitted to EEG signals in the frequency range of the tracking movements, predicted positions and velocities with moderate correlations (0.2–0.4; above chance level) in both tasks. Predictive activity in terms of decoder patterns was significant in superior parietal and parieto-occipital areas in both tasks. By contrasting the two tracking tasks, we found that predictive activity in contralateral primary sensorimotor and premotor areas exhibited significantly larger tuning to end-effector velocity when the visuomotor tracking task was performed.

## Introduction

Access to neural activity through various recording modalities allowed us to study its tuning characteristics in upper-limb movements from microscale up to macroscale levels. At the microscale level, neural spiking activity in primary motor^1^ and premotor^2^ as well as posterior parietal^3^ areas is tuned to reach direction among other movement parameters^4^. By exploiting these tuning characteristics, non-human primates^4,5^ and selected humans^6^ with spinal cord injuries have been able to control artificial end-effectors in a 3D world. At the macroscale level, in terms of non-invasively accessible neural activity, spatiotemporal tuning characteristics are not yet clearly understood with regard to upper-limb movements.

The results of functional Magnetic Resonance Imaging (fMRI) studies in humans have revealed a fronto-parietal reach network comprising dorsal premotor (PMd) and medial areas of the superior parietal lobule (SPL)^7,8^. This network is active during executed and observed reaching movements^7,8^ as well as during saccadic eye movements^9^ and exhibits directional tuning^10^. The fMRI findings, in conjunction with the successful decoding of positions and velocities from low-frequency electrocorticography (ECoG) signals^11^, suggested that information about directional movement parameters might be accessible from outside the brain. Not much later, research groups reported successful classification of reach directions^12^, and regression of end-effector positions and velocities^13^ on the basis of low-frequency magnetoencephalographic (MEG) and electroencephalographic (EEG) signals. Since then, research in this context has focused on regression of end-effector positions and velocities or classification of reach direction in center-out tasks with linear models^14^. In this paper, we focus on the regression approach.

A general limitation of studying reaching movements with a regression approach in center-out tasks is that the 2D or 3D position and velocity vectors of the end-effector point always in the same direction - the direction of the target stimulus. As a consequence, the position and velocity signals are strongly correlated during the reaching movement. For this reason, it is difficult to identify the covariate (target position, end-effector position or velocity) to which the recorded neural activity is preferentially tuned^15^. Alternatively, by studying continuous movements in a pursuit tracking task (PTT), instantaneous position and velocity can be decorrelated^15^. In a PTT, the goal is to track a moving target with an end-effector. This requires the brain to visually monitor the moving target stimulus in relation to the end-effector so that the end-effector movement can be updated to achieve the goal. In such a visuomotor (VM) task, the eyes naturally track the moving target stimulus^16^. As a consequence of this natural tracking behavior, the oculomotor network, which also spans parietal and frontal regions^17^, is activated at the same time as the reaching network^9^.

To facilitate natural behavior and isolate the neural activity related to the involvement of the upper-limb, a control condition can be introduced. In the control condition, participants would perform an oculomotor (OM) task by tracking a target stimulus only with their eyes^9,18^. In the other condition (VM task), upper-limb movement is additionally involved in the tracking. By contrasting these conditions, it should be possible to infer whether low-frequency EEG carries more information about end-effector positions and velocities during the performance of the VM or OM task, and to identify where the differences are expressed at the cortical level. We hypothesized that contralateral, primary motor and premotor areas carry more information about end-effector positions and velocities when the VM task is being performed, and that activity in areas related to the reaching and oculomotor networks is tuned to positions and velocities in both tasks.

Here, we present the tuning characteristics of low-frequency EEG activity to positions and velocities during continuous tracking movements. In two conditions, participants were asked to track a pseudo-randomly moving target either visually (OM task) or by additionally controlling a cursor with their right arm (VM task). We evaluated our approach offline by examining the recordings of healthy participants. Our experimental results confirmed that low-frequency EEG carries information about target and cursor positions and velocities in both conditions. More interestingly, when contrasting conditions, we found that the low-frequency EEG carried more information about the instantaneous cursor velocity during the VM task rather than during the OM task. The differences were mainly reflected in the premotor and contralateral primary sensorimotor areas. The temporal tuning characteristics of these differences indicated that the predictive neural activity preceded cursor velocity with 150 ms. Therefore, we could show that low-frequency EEG activity, originating in premotor and primary sensorimotor areas, can at least offline be used to predict the velocities of executed upper-limb movements.

## Results

**Figure 1:**
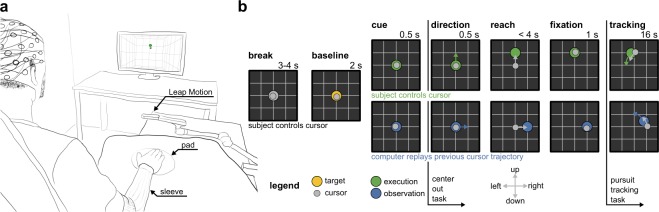
Experimental setup and paradigm. (**a**) Participants sat in a comfortable chair positioned 1.4 m from a computer screen. Both arms were supported at the same height. The right arm rested on a table at a comfortable position. The friction between arm and surface was reduced by a sleeve and a circular pad positioned between hand and table. Palm position movements were recorded by a LeapMotion controller (LeapMotion Inc., USA) located 20 cm above the hand. Forward/backward hand movements on the table were mapped to upward/downward cursor movements on the screen. (**b**) Each trial started with a 3–4 s break during which the target (large ball) resided in the center. A 2 s baseline period was initiated when the target turned yellow. During this period, participants were asked to keep their hand in the resting position and, thereby, the cursor in the center of the screen. A visual cue indicated the condition, either execution (green target) or observation (blue target), followed by a center-out task in four directions. The direction was indicated by the target movement (0.5 s duration; arrows visualize movement in the individual images). After 1 s of fixation, a pursuit tracking task was performed for 16 s. A colored target stimulus (yellow, green, or blue) instructed the participants to fixate and track the target with their eyes. In the execution condition the participants controlled the cursor, while in the observation condition, the computer replayed a previously executed cursor trajectory which matched to the current target trajectory. See Supplementary Video S1 for examples.

To test our hypotheses, we recorded high-density EEG and electrooculography (EOG) from 15 healthy participants during a two-dimensional PTT. In every trial, the PTT was preceded by a short, visually guided, center-out reaching task. Here, we present our findings during the PTT. Figure 1 depicts the experimental setup and paradigm. The paradigm separated two conditions. In the first condition (execution, VM task), participants were asked to track a pseudo-randomly moving target with their gaze and right hand by manipulating a cursor (Fig. 1a). In the second condition (observation, OM task), participants were asked to track the moving target with their gaze, while keeping their right hand in a resting position. To obtain similar visual input and tracking dynamics in both conditions, we replayed the participant’s most recent, matching, executed cursor trajectory in observation condition trials. All results presented subsequently were determined after pre-processing, correcting for EOG artifacts^19^ and downsampling the recorded data to 10 Hz (see Methods). Throughout the text, grand average results are presented in the form of the mean value and its standard error.

### Tracking analysis

**Figure 2:**
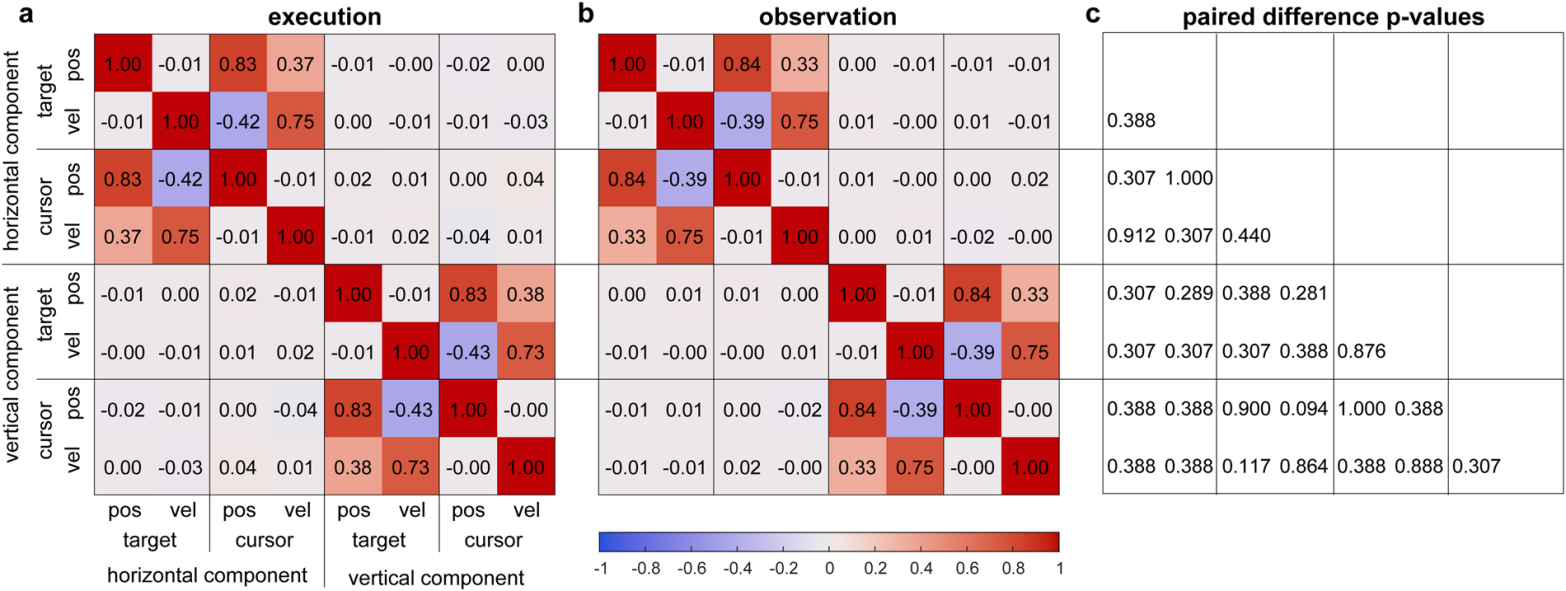
Group-level stimuli cross-correlations in both conditions. (**a**) Cross-correlations between the two-dimensional movement parameters (target position, target velocity, cursor position, cursor velocity) in execution condition. (**b**) Cross-correlations in observation condition. (**c**) *P*-values for paired Wilcoxon sign rank tests between conditions. *P*-values were adjusted^55^ for 28 comparisons to control the false discovery rate (FDR) at a level of 0.05.

To analyze the tracking dynamics, we computed cross-correlations between the positions and velocities of both stimuli in execution and observation conditions. Figure 2a summarizes the grand average cross-correlations in the execution condition. The large cross-correlations (*r* > 0.7) observed between signals of the same movement parameter (e.g, target and cursor position) show that participants complied with the instruction to minimize the distance to the target. Cross-correlations between the position and velocity of the same stimulus were negligible (*|r*| < 0.02; e.g, target position and target velocity), while we observed moderate (|*r*| ~ 0.4; e.g, target position and cursor velocity) cross-correlations across stimuli. Our target trajectory generation procedure ensured decorrelated horizontal and vertical components. Hence, cross-correlations observed with any signal from the other component were negligible (|*r*| < 0.05). Figure 2b summarizes the grand average cross-correlations in observation condition. We did not detect significant differences to the execution condition results (Fig. 2c).

**Figure 3:**
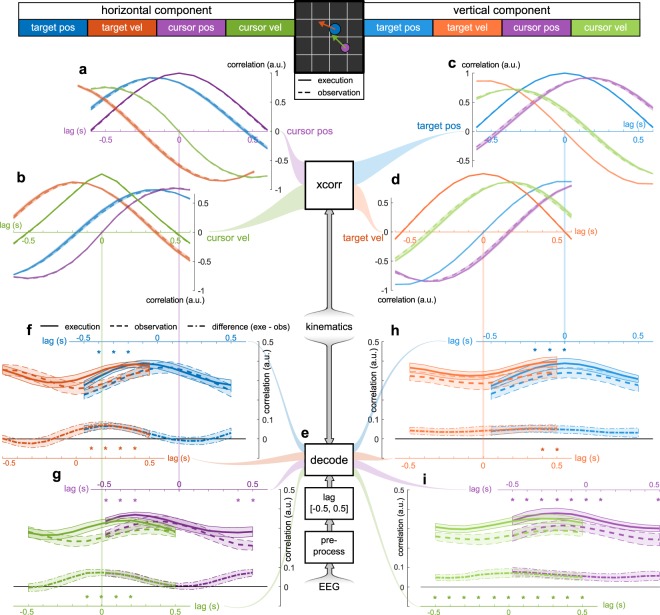
Grand average movement parameter auto-/cross-correlation curves, and movement parameter tuning curves. (**a–d**) Grand average stimuli auto- and cross-correlations. (**a**) Auto- and cross-correlation curves of horizontal components relative to the horizontal cursor position during execution (solid lines) and observation (dashed lines). Movement parameters are color-coded. Shaded areas represent the standard-error of the mean. Cross-correlations were evaluated for lags, ranging from −0.5 s (leading relative to cursor position) to 0.5 s (lagging) in 0.1 s steps. (**b**) Auto- and cross-correlation curves of horizontal components relative to the horizontal cursor velocity. (**c)**, Auto- and cross-correlation curves of vertical components relative to the vertical target position. (**d**) Vertical target velocity. (**e**) Outline of the regression approach. After EEG preprocessing (including bandpass-filtering), a sliding window (one sample) was used to decode the movement parameters at different lags. (**f**–**i**) Grand average correlations between movement parameters and their estimates at different lags (tuning curves). (**f**) Tuning curves for the horizontal target position (blue) and velocity (orange). The mean and its standard error summarize the results for execution (solid lines), observation (dashed lines) and their paired difference (dash dotted lines). Cross-correlation peaks between target position and velocity were used to align the time-lag axes. Lags with significant differences between conditions (paired Wilcoxon sign-rank tests, FDR adjustment for 88 comparisons, 0.05 significance level) are highlighted (*). (**g**) Tuning curves for horizontal cursor position (violet) and velocity (green). (**h**) Tuning curves for vertical target position (blue) and velocity (orange). (**i**) Tuning curves for vertical cursor position (violet) and velocity (green).

To estimate the temporal dependencies among the four movement parameters (target position, target velocity, cursor position, cursor velocity) per component, we computed cross-correlations over lags in the interval [−0.5 s, 0.5 s] in steps of 0.1 s. Figures 3a–d depict the results for the horizontal (a,b) and vertical (c,d) components. We aligned the individual figures based on the peak cross-correlations between pairs of movement parameters. For example, the origin in Figure 3b is shifted by −0.525 s compared to that in Figure 3a because the cursor velocity was maximally correlated with cursor position 0.525 s in the future. In the execution condition, the participants reacted with their hand movements (cursor trajectories) to the pseudo-random target trajectories. This means that the properties of the target trajectories (e.g, cross-correlation peak between target position and velocity) also determined the properties of the cursor trajectories.

The cross-correlation peak between target and cursor position can be used to infer information about the participants’ tracking behavior. We used the lag of the cross-correlation peak to estimate the latency between the target and cursor. In the execution condition, the latency reflected the duration that a participant took to adjust the cursor movement to the pseudo-random target movement. The cross-correlation between the target and cursor position peaked at a delay of 153 ± 19 ms at group level. After accounting for a 55 ± 1 ms delay, introduced by our online processing system which transformed hand movements into cursor movements, the average latency of hand movements was approximately 100 ms. This result is in accordance with the findings of behavioral studies, which report that a minimum latency of 80–100 ms is needed for a visual or proprioceptive signal to influence an ongoing movement^20,21^.

### Movement parameter tuning curves

We estimated tuning curves for each movement parameter with a single sample, sliding-window, linear regression approach^13,22^. The regression approach is outlined in Figure 3e. At different lags, a partial least squares (PLS)^23^ estimator was used to decode each movement parameter from the EEG within the sliding window (one sample). This modelling approach implied that the relevant activity in the signal used for decoding (EEG) has to be in the same frequency range as the signal to be decoded (e.g, horizontal cursor velocity)^24^. To extract the relevant activity in the frequency range (0.3 to 0.6 Hz) of the target and cursor trajectories, we bandpass-filtered the EEG (Supplementary Fig. S3 shows power spectral densities of the bandpass-filtered EEG and the movement parameters). In a cross-validation scheme, we computed correlations between the signals to be decoded (e.g, horizontal cursor velocity) and their estimates for each lag to generate the tuning curves.

Figures 3f–i summarize the grand average tuning curves for both conditions. Due to the independence between the horizontal and vertical components (Fig. 2), the tuning curves in Figures 3f–i are complementary. For both components, the grand average correlations ranged from 0.2 to 0.4. We used a shuffling approach to estimate chance levels for each participant. The chance levels were similar across components, conditions and lags (target position *r*_*chance*_ = 0.13 ± 0.003, target velocity *r*_*chance*_ = 0.12 ± 0.002, cursor position *r*_*chance*_ = 0.12 ± 0.003, cursor velocity *r*_*chance*_ = 0.10 ± 0.002). Compared to chance level, the observed correlations were significant for all participants, components, conditions, movement parameters and lags. As in Figures 3a–d, we aligned the tuning curves (Fig. 3f–i) according to the peak cross-correlations between pairs of movement parameters. After the alignment, we observed three effects.

As a first effect, we found that in the observation condition the tuning curves in Figures 3f–i (dashed lines) were modulated by the auto-/cross-correlation with target position. That is, an increase in the tuning curve of a movement parameter coincided with an increase in the absolute auto-/cross-correlation between the movement parameter and the target position signal. We observed this effect for all movement parameters due to the dependencies between them. The dependencies are reflected in the auto-/cross-correlation curves (Fig. 3a–d). For example, the tuning curve of the vertical target position (Fig. 3h, blue dashed line) exhibited a similar waveform compared to the target position’s autocorrelation curve (Fig. 3c, blue line). The tuning curve of the horizontal cursor position (Fig. 3g, violet dashed line) and its cross-correlation curve with the target position (Fig. 3a, blue line) represents another example. The size of the effect was approximately 0.1 for both components and maximal for target position. In the execution condition (solid lines), we detected the same modulation. However, it was partially masked by the other effects.

The second effect observed concerns the vertical component (Fig. 3h,i). The paired differences between execution and observation conditions (dash-dotted lines) exhibited a positive effect on all movement parameters and lags. That is, in the execution condition, the low-frequency EEG contained significantly more information about the movement parameters of the vertical component. The effect was largest for vertical cursor position and velocity with an average difference in correlation of 0.05 (Fig. 3i, violet and green dash-dotted lines).

The third effect concerned the differences in tuning curves for both components (Fig. 3f–i, dash-dotted lines). The differences were modulated by the absolute auto-/cross-correlation between a movement parameter and cursor velocity (Fig. 3a–d, green lines). The effect was prominent for the horizontal component and largely masked by the second effect for the vertical component. For example, the difference in tuning curves for the horizontal cursor position (Fig. 3g, violet dash-dotted line) resembled the absolute value of its cross-correlation curve with the horizontal cursor velocity (Fig. 3a, green line). The size of the effect was maximal (approx. 0.07) for the horizontal cursor velocity at lag 0 (Fig. 3g, green dash-dotted line). Taken together, we inferred that the extracted EEG carried significantly more information about the instantaneous (lag 0 s) cursor velocity in the execution condition.

We were also interested in assessing which brain areas encoded more information in the execution condition than in the observation condition. To determine which brain areas contributed to the third effect, we selected the cursor velocity decoder models at a lag of 0 s as representatives and computed their associated activation patterns^25^. The patterns were subsequently mapped to the cortical surface by applying EEG source imaging^26,27^ on a template head model. In source space, we computed pairwise differences between the conditions for the Euclidean norm of each voxel (see Methods).

**Figure 4:**
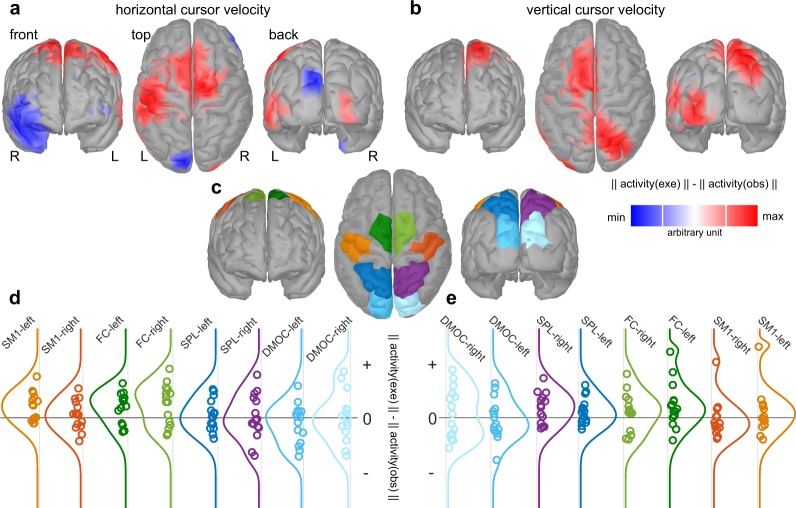
Grand average pattern activity difference between conditions in source space for single-lag (lag 0) cursor velocity decoder models. (**a**) Horizontal cursor velocity pattern. Voxel color indicates the sign of the difference in norms; positive (red) indicates larger pattern activity in execution. Voxels with a difference in norms less than half of the absolute maximum are shaded with gray to emphasize the sites with the largest effects. (**b**) Vertical cursor velocity pattern. (**c**) Anatomical regions of interest (ROI)s, covering dorsomedial occipital cortex (DMOC), superior parietal lobule (SPL), fronto-central (FC) and primary sensorimotor areas (SM) of both hemispheres. (**d**) Density estimates of the differences in ROI activity for participants for the horizontal cursor velocity. Each point represents one participant. Density curves follow the ROI color-coding scheme. (**e**) As in (**d**) for vertical cursor velocity.

Figure 4 depicts the grand average difference in pattern norms for the horizontal (Fig. 4a) and vertical (Fig. 4b) cursor velocities at lag 0. We defined eight anatomical regions of interest (ROIs) to span areas related to the fronto-parietal reaching network^7,9^. They are dorsomedial occipital cortex (DMOC), superior parietal lobule (SPL), fronto-central (FC) and primary sensorimotor areas (SM) of both hemisphere (Fig. 4c). We summarized the pattern activity of each ROI as the average of its voxels. Figures 4d,e depict the distribution of both horizontal and vertical components for all participants. Regarding the horizontal component (Fig. 4d), we observed a positive effect in FC and left SM areas. For the vertical component (Fig. 4e), we observed positive effects in right SPL and both FC areas. Considering their positive sign, the results indicate that the activity in the areas contained more information about the instantaneous cursor velocity in execution condition.

**Figure 5:**
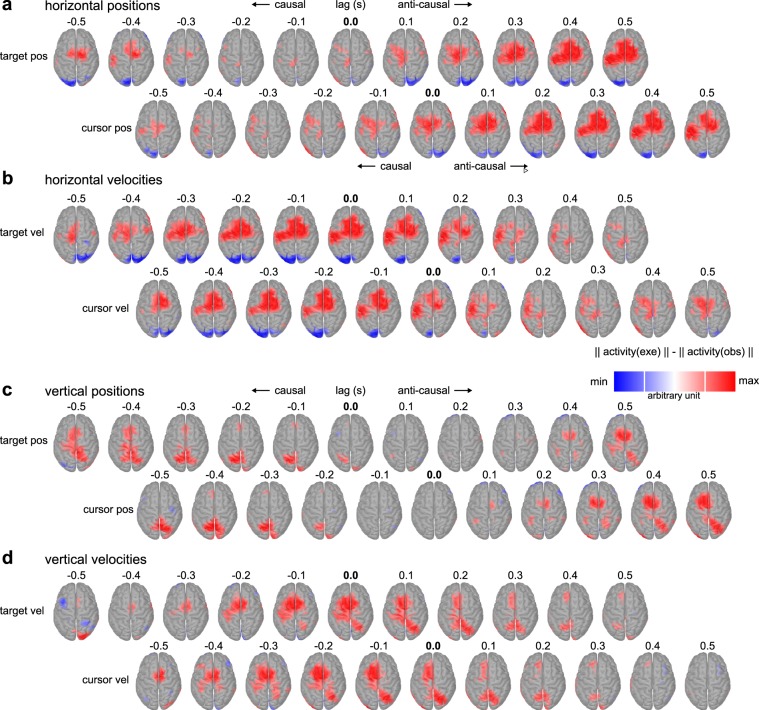
Grand average pattern activity differences between conditions for all single-lag decoder models. (**a**) Single-lag decoder patterns for the horizontal target (top) and cursor (bottom) positions for lags ranging from −0.5 s (brain activity leading relative to the position signals) to 0.5 s (brain activity lagging). As before, cross-correlation peaks between the target and cursor positions were used to align the time-lag axes. The red color indicates larger voxel activity in the execution condition. (**b**) Single-lag decoder patterns for the horizontal target (top) and cursor (bottom) velocities. (**c**) As in (**a**) for the vertical positions. (**d**) As in (**b**) for the vertical velocities.

In Figure 5 we show the difference in pattern norms for all single-lag models (Fig. 3f–i), to demonstrate how the differences in tuning curves are reflected on the cortical surface. Negative lags indicated leading brain activity (causal tuning), while positive lags indicated lagging brain activity (anti-causal tuning). The difference in activation patterns in fronto-central and contralateral sensorimotor areas was tuned to the horizontal and vertical cursor velocity in the [−0.5, 0.1] s interval and peaked around −0.1 to −0.2 s (Fig. 5b,d; bottom rows). As before, due to the temporal dependence between the position and velocity signals (Fig. 3a–d), we also observed tuning effects for the position signals. The difference in activation patterns was anti-causally tuned to cursor position for lags in the range [0, 0.5] s (Fig. 5a,c; bottom rows). Overall, the strength of the differences was more pronounced for the horizontal component. Similar to the cursor velocity pattern at lag 0 (Fig. 4b,e), we observed a positive effect in SPL only on the vertical component movement parameters (Fig. 5c,d). With respect to vertical cursor velocity (Fig. 5d; bottom row), the positive effect started in the right SPL at lag −0.3 s, peaked at lag 0 s, subsequently translated to the left SPL and faded at lag 0.3 s.

### Multiple-lag cursor velocity prediction

To exploit the tuning of neural activity over multiple lags, we extended the feature set by using multiple samples in the sliding window, linear regression approach. We evaluated sliding windows covering the samples at lags [−0.1, 0] s to [−0.5, 0] s in 0.1 s steps to predict the horizontal and vertical cursor velocities. The correlations between the recorded and decoded cursor velocities initially increased, but became saturated for windows exceeding [−0.3, 0] s (Fig. S4). For the [−0.3, 0] s window, the grand average test set correlations were *r*_*exe*_ = 0.40 ± 0.02, *r*_*obs*_ = 0.36 ± 0.04 for the horizontal component and *r*_*exe*_ = 0.41 ± 0.03, *r*_*obs*_ = 0.33 ± 0.03 for the vertical component.

**Figure 6:**
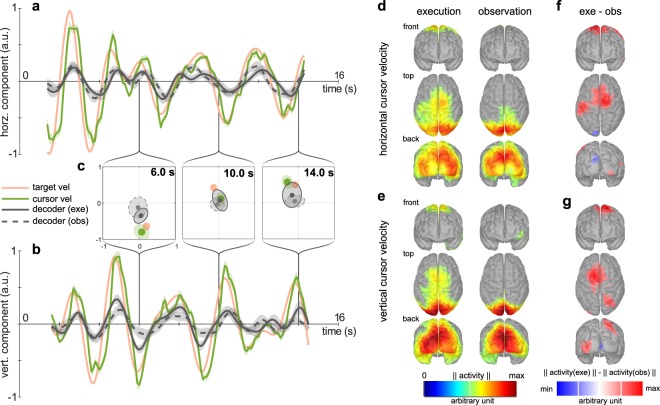
Grand average cursor velocity prediction for a [−0.3, 0] s estimation window. (**a–c**) Illustrations of executed and decoded cursor velocities for a specific target trajectory. (**a**) Grand average horizontal target (orange line), cursor (green line) and decoded cursor velocity in execution (gray solid line) and observation (gray dashed line) conditions. Shaded areas summarize the standard error of the mean. (**b**) As in (**a**) for the vertical component. (**c**) 2D representation for single time points. Dots indicate the group-level average. Dispersion over participants is summarized by the square root of the covariance matrix. (**d–g**) Grand average multiple-lag decoder patterns. (**d**) Horizontal cursor velocity patterns in the execution (left) and observation (right) conditions. Pattern activity norms were averaged over lags. The voxel color indicates strength of activity. (**e**) Vertical cursor velocity patterns in the execution (left) and observation (right) conditions. (**f**) Difference between lag-averaged pattern norms for the horizontal component. The voxel color indicates the sign and strength of the difference in the pattern activity. (**g**) As in (**f**) for the vertical component.

To visualize the decoded cursor velocities, we selected a representative trajectory and summarized the results over participants. Figures 6a–c show the recorded target, cursor and the decoded cursor velocities for this particular trajectory in both conditions. The small standard-error around the recorded cursor velocities (green shaded area) demonstrates that the participants were tracking the target consistently. Compared to the recorded cursor velocities, the decoded cursor velocities exhibited more variance over participants than their neural predictions (Fig. 6c). Still, the grand average decoded cursor velocities were strongly correlated for both components and conditions. The grand average correlations were *r*_*exe*_ = 0.83 ± 0.02, *r*_*obs*_ = 0.82 ± 0.02 on average for the 90 trajectories on the horizontal component, and *r*_*exe*_ = 0.85 ± 0.02, *r*_*obs*_ = 0.80 ± 0.03 on the vertical component. This reflects a 0.40 gain in correlation at the group level compared to the results at participant level.

As before, we computed activation patterns and projected them to the cortex. Figures 6d,e depict the grand average patterns (average over participants and lags), and Table 1 lists the *p*-values of non-parametric permutation paired *t*-tests for the eight ROIs. Compared to chance level, the pattern activity was significant in DMOC areas in both conditions. SPL pattern activity was significant in the execution and mainly in the observation condition; the effect on vertical cursor velocity did not reach significance in observation condition. FC pattern activity was generally larger in the execution condition (Fig. 6f,g). The differences observed between execution and observation conditions were in line with the single-lag results (Fig. 3f–i). They were significant in right FC and left SM for the horizontal component, and in right SPL for the vertical component. The effects on left FC (and right FC for the vertical component) did not reach significance.

**Table 1:** Significance of ROI activation for multiple lag cursor velocity decoders (exe vs. shuffled exe, obs vs. shuffled obs, and exe vs. obs).

movement parameter	condition	ROI							
		DMOC right	DMOC left	SPL right	SPL left	FC right	FC left	SM1 right	SM1 left
horizontal cursor vel	exe	**0.0270**	**0.0096**	**0.0373**	**0.0096**	**0.0373**	0.13	0.49	0.15
	obs	**0.0096**	**0.0096**	**0.0096**	**0.0120**	0.32	0.36	0.79	0.30
	exe - obs	0.79	0.57	0.60	0.60	**0.0224**	*0.067*	0.25	**0.0148**
vertical cursor vel	exe	**0.0096**	**0.0120**	**0.0096**	**0.0096**	*0.068*	0.11	0.74	0.72
	obs	**0.0096**	**0.0096**	*0.068*	*0.094*	0.79	0.14	0.79	0.53
	exe - obs	0.82	0.49	**0.0171**	0.36	*0.098*	*0.051*	0.65	0.51

*P*-values were computed with two-sided non-parametric permutation paired *t*-tests (1000 permutations). Significant differences (FDR adjustment of *p*-values for 48 comparisons, 0.05 significance level) appear in bold text.

### Visual tracking analysis

We examined the EOG signals to compare the visual tracking behavior between conditions by computing cross-correlations between the horizontal and vertical target position and the associated EOG derivative signals. The cross-correlations peaked at lag 0 for both conditions, indicating that the participants’ gaze was focused on the target’s instantaneous position. By comparing the correlation values at lag 0, we detected significant differences among conditions and components (significance levels were Bonferroni corrected from 0.05 to 0.01 for 5 two-sided paired Wilcoxon sign-rank tests). We found a slightly lower degree of correlation in the execution condition compared to the observation condition for the horizontal component (*r*_*exe*_ = 0.88 ± 0.01, *r*_*obs*_ = 0.90 ± 0.02, *p* = 0.00537), while the degree of correlation was higher in the execution condition for the vertical component (*r*_*exe*_ = 0.79 ± 0.02, *r*_*obs*_ = 0.70 ± 0.04, *p* = 0.00153). Within conditions, the degrees of correlation were higher for the horizontal component (*p*_*exe*_ = 0.00012; *p*_*obs*_ = 0.00006).

In the execution condition, the VM task required the processing of visual feedback about the cursor in relation to the moving target, while in the observation condition, the cursor was not task-relevant. Authors of previous behavioral studies have reported a reduced blink rate (BR) if more visual information was processed^28,29^. In our study, we detected blinks by thresholding the vertical EOG derivative^19^. As predicted, we found a significantly lower BR in terms of blinks per second (bps) in the execution condition as compared to the observation condition (*BR*_*exe*_ = 0.019 ± 0.006 *bps*, *BR*_*obs*_ = 0.028 ± 0.006 *bps*, *p* = 0.0015).

## Discussion

We have presented a novel paradigm, which was tailored to study the tuning characteristics of human, low-frequency EEG to target and cursor (end-effector) positions and velocities in the presence of eye movements. Our paradigm allowed us to distinguish between two conditions with similar tracking dynamics, but with different cursor-control origin. By not inhibiting eye movements during the PTT, we could study tracking movements in a natural fashion and focus on the effects related to the involvement of the upper limb. We presented evidence that this involvement indeed influences the spatiotemporal expression of information about end-effector positions and velocities in the low-frequency EEG activity.

In a PTT, participants typically manipulate an end-effector to minimize its distance to a target. Typically, task compliance results in high cross-correlations between movement parameters of the same type (e.g, position). However, the cross-correlations between positions and velocities depend on the properties of the target trajectories. We created a trade-off between task difficulty, bandwidth and steepness of the increase in correlation over lags. By using the 0.3 to 0.6 Hz band, we could study EEG in a similar frequency range as those examined in previous studies^13,22^, and shift the peak in cross-correlation between target velocity and position to 0.55 s. After accounting for the dependence between the movement parameters by aligning the tuning curves, we determined one effect in both conditions and two effects in the difference between conditions.

The effect observed in both conditions and components concerned the modulation of the tuning curves by the amount of cross-correlation between a movement parameter and target position. This effect let us to infer that information about the instantaneous target position was encoded in the low-frequency EEG. In the observation condition, the effect was prominent, while it was partially masked by the other effects in the execution condition. The target position was particularly relevant during the PTT. In both conditions, the participants had to keep their gaze fixated on the target. The fact that a peak in the correlation between target position and EOG derivatives occurred at lag 0 confirmed that the participants were able to accomplish the task. This finding is in accordance with findings for human smooth pursuit behavior for a bandlimited pseudo-randomly moving stimulus^30^. As a consequence, eye movement artifacts were also phase locked to the target position signal. To assess which sources contributed to the observed effect, we computed patterns for target position decoders at lag 0 and projected them to source space (Fig. S5). The grand average patterns for both conditions indicated that the contributions originated from a combination of brain activity with the largest predictive activity in parieto-occipital areas and residual eye artifacts.

As with the modulation of the tuning curves with target position in both conditions, we observed a modulation of the differences between conditions with cursor velocity. By mapping single-lag cursor velocity model patterns to source space and computing differences between conditions, we observed a stronger effect during the execution condition in both FC and contralateral SM ROIs (Fig. 5). The FC ROIs covered dorsal premotor (PMd) and supplementary motor areas (SMA). Their involvement in reaching is in accordance with the findings of imaging studies in humans^31^. The difference in activation patterns in FC and contralateral SM ROIs was sustained over multiple lags, with its peak activity leading cursor velocity by approximately 150 ms. The delay can be reduced to about 95 ms by accounting for the 55 ms delay between the hand and cursor movement, introduced by the online processing system. The remaining 95 ms could be explained by motor output delays^32^. Estimating the actual motor output delay is not a straightforward task, since it depends on the task demands and the type of perturbation among other factors^32^. However, Paninski *et al*. studied the tuning of movement parameters in a comparable PTT^15^. They investigated M1 single unit activity in non-human primates and reported that neural activity was tuned to cursor velocity in a [−400, 400] ms lag range (peak at −100 ms; neural activity leading). Thus, a stronger degree of tuning of neural activity to cursor velocity in motor areas during the execution condition offers a plausible explanation. An alternative explanation would be offered by anti-causal tuning to the cursor position for lags in the range [0, 500] ms (Fig. 5a,c). Tuning to the cursor position peaked at 300 to 400 ms, which would reflect feedback processing (neural activity lagging). Experimental results on decoding movement parameters in behaving non-human primate spiking activity^1,4^ and local field potentials^33^, together with results of studies on human MEG^34^ and ECoG^35^, show that tuning of SM activity to movement parameters yields peaking activity around 100 ms before the movement. Taken together, the differences in the activation patterns reflects more likely information about upcoming cursor velocities. Consequently, the observed effects for the cursor positions can be explained by the cross-correlations between the movement parameters (Fig. 3a–d).

The third effect observed concerned the vertical component alone. In the execution condition, we observed that the correlations of the tuning curves were generally higher (Fig. 3h,i), a 0.1 higher correlation between vertical EOG derivative and target position and a decrease in blink rate. Moreover, the activation patterns were significantly stronger in the SPL (Fig. 5b,d, Table 1). Relative decreases in the blink rate have been shown to be related to the processing of more visual information^28^ and more demanding tasks^29^. This reflects the difference between visuomotor (VM) and oculomotor (OM) tasks studied here. Behavioral and decoding results combined indicate greater engagement in tracking vertical component signals in the VM task. We offer two non-exclusive explanations for this phenomenon. First, unlike the horizontal component, the vertical component mapping was not congruent; this means that forward hand movements were mapped to upward cursor movements. Therefore, the increase in SPL activity could be explained by the integration of incongruent proprioceptive and visual information. Second, we studied two stimuli, moving in two uncorrelated dimensions, which meant that the oculomotor system had to keep track of both dimensions. The findings of behavioral studies^30^ and our results show that smooth pursuit is more accurate for the horizontal component. Accurate control of the upper-limb in the VM task could require the visual system to extract more information about the vertical component and, as a side effect, improve smooth pursuit. Since the SPL is involved in smooth pursuit control^36^, the increase in information about the vertical component could be explained.

By combining multiple lags to predict cursor velocities we could raise grand average decoder correlations by around 0.05 to 0.4 in the execution condition and to 0.35 in the observation condition. These correspond to correlations reported in previous EEG decoding studies in center-out^13^ and continuous movement tasks^37,38^. By averaging over participants, the correlations improved drastically to 0.8. The 2D plots in Figure 6c illustrate the reason for this effect. The grand average decoded cursor velocity is frequently in the same quadrant as the recorded cursor velocity; however, the variance among participants is considerable. Thus, the individual correlations are substantially lower. We inferred that the signal to noise ratio could be drastically improved by averaging the response over participants and consequently that the low-frequency EEG strongly correlates with positions and velocities at the group level in both conditions.

The grand average multiple lag cursor velocity decoder model patterns (Fig. 6d,e) demonstrate that the contributing sources were primarily of cortical origin in both conditions. Therefore, it is unlikely that the cursor velocity decoders relied on residual eye movement artifacts. It is also unlikely that arm or neck movement artifacts contributed, considered that there was no arm movement in the observation condition, and that the difference in decoder patterns (Fig. 6f,g) were primarily located in contralateral primary sensorimotor, fronto-central and parietal areas. In both conditions and in both components, pattern activity was strongest in the parieto-occipital and parietal areas (Fig. 6d,e). The associated DMOC and SPL ROIs showed significantly stronger pattern activity compared to the patterns of shuffled data (Table 1). This is in accordance with an increase in blood oxygenation level dependent (BOLD) activity in these areas during executed and observed reaching movements^39^. Moreover, the strong tuning of parieto-occipital and parietal areas in both conditions, reported here, is in accordance with the modulation of BOLD activity by movement direction in an fMRI adaptation study^10^. Since there was no significant difference in the DMOC ROIs between conditions (Table 1; exe - obs), we inferred that the predictive activity in the parieto-occipital areas was not specific to the VM task. That is, the predictive activity in parieto-occipital areas did not require the involvement of the upper-limb.

In conclusion, we demonstrated that low-frequency EEG carries information about target and cursor positions and velocities, which is primarily encoded in fronto-parietal and parieto-occipital networks. By contrasting between the decoder patterns of the VM and OM tracking tasks, we found that the degree of tuning in fronto-central and contralateral primary sensorimotor areas to the instantaneous cursor velocity was significantly larger in the VM tracking task. The temporal tuning characteristics indicate that neural activity lead cursor velocity by approximately 150 ms (hand velocity by 95 ms). Altogether, the presented results on spatial and temporal tuning characteristics of the low-frequency EEG extend the findings of previous decoding studies. Moreover, we believe that it is possible to transfer our findings to individuals with tetraplegia, since the participants in this study moved their right arm only during the VM task, but the decoder correlations were clearly above chance level in both tracking tasks. Future closed-loop studies need to investigate whether the tuning characteristics of low-frequency EEG can be exploited to control an end-effector and whether the control skill can be improved.

## Methods

### Participants

Fifteen people, aged 23.8 ± 0.8 years, participated in this study. All received payment to compensate for their participation. Nine of the participants were female. All participants self-reported to have normal or corrected-to-normal vision and to be right handed. Eleven participants had previously participated at least once in an EEG experiment. All signed an informed consent form after they had been instructed about the purpose and procedure of the study. The experimental procedure conformed to the declaration of Helsinki and was approved by the ethics committee of the Medical University of Graz (approval number 29-058 ex 16/17).

### Experimental set-up

Figure 1a depicts the recording environment. Participants sat in a shielded room, positioned 1.4 m away from a computer screen. Their left arm was supported by an arm rest, while the right arm was supported by a planar surface at the same height. To reduce friction between the right arm and the surface, participants were asked to wear a sleeve and place their hand on a circular pad. A LeapMotion controller (LeapMotion Inc., USA), placed 20 cm above the hand, was used to record the right hand’s palm position. After participants found a comfortable resting position, the right hand’s palm position was mapped to the origin (center of the screen) in the virtual environment. In analogy to the interaction with a computer by using a computer mouse, we decided to map rightward/forward hand movements to rightward/upward cursor movements. In order to create a trade-off between movement range and movement/muscular artifacts in the EEG, we mapped a circle with a 5 cm radius around the resting position to a circle with a 16 cm radius on the screen. The limits of the circle on the screen were indicated by the bounds of a virtual grid. E.g. by moving their hand 5 cm to the right, the participants could make the cursor touch the grid on the right side.

### Experimental procedure

The experimental procedure consisted of 4 blocks, lasting 3 hours in total. In the first block, participants were asked to familiarize themselves with the paradigm (approx. 10 min). In the second and fourth block, eye artifacts (blinks and eye movements) and resting activity were recorded for 5 min. The detailed procedure is described in^19^. In the third block, participants performed the main experimental task according to the paradigm illustrated in Figure 1b. Each trial implemented a center-out reaching task followed by a PTT. A yellow target stimulus marked the beginning of a trial. It triggered the participants to fixate their gaze upon the target. The paradigm distinguished between two conditions. In the observation condition (blue target), participants merely tracked the moving target visually while the computer replayed a previous cursor trajectory. In the execution condition (green target), participants additionally had to minimize the distance between the target and cursor by moving their right hand and thereby the cursor. A total of 180 trials (90 per condition, pseudo randomly distributed) were recorded in 20 runs with short breaks in between. We additionally recorded 180 short trials (90 per condition, pseudo randomly distributed with the other trials within the 20 runs). A short trial ended after the center-out task. The data recorded during short trials were not used in this analysis. Supplementary Movie S1 shows the tracking behavior of representative participants in both conditions during long (center-out + PTT) and short (center-out) trials.

Target trajectories were generated offline and were identical across participant. Twelve base target trajectories were sampled from pink noise, which was band-passed in the frequency range of 0.3 to 0.6 Hz according to the procedure described by Paninski *et al*.^15^. We sampled the horizontal and vertical components independently so that they were uncorrelated. The trajectory pool was extended by adding rotated (90°, 180° and 270°) and mirrored versions of the base target trajectories. This yielded a total of 96 trajectories; 90 of these were randomly distributed over the 180 trials (once per condition). This procedure ensured uncorrelated position and velocity signals at lag 0 (Fig. S1).

The results of pilot studies revealed that the tracking dynamics varied among participants and over time. To achieve similar and participant specific tracking dynamics between the conditions, we implemented an adaptive approach. In observation condition trials, the most recent cursor trajectory of all matching versions (original, rotated and/or mirrored) of the associated base target trajectory was selected for replay. Details about the cursor trajectory replay procedure are described in the supplementary methods.

### Data recording and pre-processing

All data was recorded using the labstreaming layer (LSL) protocol (https://github.com/sccn/labstreaminglayer). 64 active electrodes (actiCAP, Brain Products GmbH, Germany) were placed on the scalp according to the 10–10 system. The reference and ground electrodes were positioned at the right mastoid and AFz. Six additional active electrodes were placed at the superior, inferior and outer canthi of the right and left eyes to record EOG. Figure S2 visualizes the locations of all 70 electrodes. EEG and EOG data were recorded at 1 kHz (BrainAmp, Brain Products GmbH, Germany). The paradigm was implemented in Python 2.7 based on the simulation and neuroscience application (SNAP) platform (https://github.com/sccn/SNAP) and the 3D engine Panda3D (https://www.panda3d.org). The screen position signals of the visual stimuli (cursor, target) were recorded via LSL at 60 Hz and synchronized offline with the EEG signals by means of a photodiode, which captured an impulse on the screen at the start of each trial. All signals were then resampled to 200 Hz.

**Figure 7:**
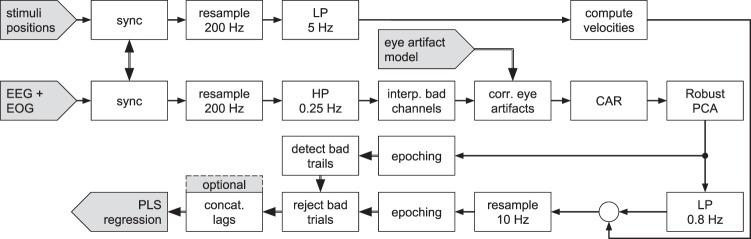
Signal pre-processing pipeline. After synchronization, brain signals were resampled, high-pass filtered and bad channels were spherically interpolated. Then, eye artifacts were attenuated^19^, followed by a conversion to a common average reference. Next, Robust PCA^41^ was applied to attenuate single electrode outliers. A subsequent low-pass filter was applied to extract the EEG signals in the frequency range of the target and cursor movements. Stimuli position signals were low-pass filtered before computing velocities and then concatenated to the EEG. After epoching, marked trials were rejected. Samples were optionally concatenated to extend the feature space for PLS regression.

The pre-processing pipeline is depicted in Figure 7 and was implemented in Matlab (Matlab 2015b, Mathworks Inc., USA) and the open source software EEGLAB^40^ version 14.1.1. EEG data were high-pass filtered (0.25 Hz cut-off frequency, Butterworth filter, eighth order, zero-phase). Data cleaning was initiated by a spherical interpolation of channels with poor signal quality (visual inspection). We interpolated 2.1 channels on average (Table S1). Eye movements and blinks were attenuated by applying the artifact subspace subtraction algorithm (outline in subsection *eye artifact correction*). The EEG channels were subsequently converted to common average reference (CAR). We then applied robust principal component analysis (Robust PCA)^41^ to attenuate occasional electrode pops and low-frequency drifts. The motivation behind Robust PCA is to separate a data matrix **X** (raw EEG) into a sum of a low rank matrix **L** (EEG) and a sparse matrix **S** (occasional single or few electrode outliers, e.g, pops). The optimization problem can be formulated as

1 $${\rm{\min }}\,{\Vert {\bf{L}}\Vert }_{\ast }+{\rm{\lambda }}\,{\Vert {\bf{S}}\Vert }_{1}\,{\rm{s}}.{\rm{t}}.\,{\bf{X}}={\bf{L}}+{\bf{S}}$$

and solved iteratively^41^. We fixed the regularization parameter ${\rm{\lambda }}=\frac{1.5}{\sqrt{{\rm{N}}}}$ with N being the number of samples. All subsequent processing steps were applied to the extracted low rank data matrix **L**. We epoched the data into 14 s trials, starting 1 s after tracking onset. Trials were marked for rejection if (1) the EEG signal of any channel exceeded a threshold of +/−200 µV or had an abnormal probability or kurtosis (more than 6 standard deviations beyond the mean), (2) the correlation of any EOG derivative (HEOG/VEOG) with the target position (horizontal/vertical) was improbable (more than 4 standard deviations beyond the mean), and (3) if a tracking error appeared (i.e, if hand tracking was lost or jerky). We applied the joint probability and kurtosis rejection criteria twice to detect gross outliers in the first iteration, and subtle outliers in the second iteration. All criteria combined marked an average of 16% of the trials for rejection. Supplementary Table S1 lists detailed information for each participant. Before actually rejecting trials, a low-pass filter (0.8 Hz cut-off frequency, Butterworth filter, fourth order, zero-phase) was used to extract EEG signals in the frequency of the target and cursor movements.

Stimuli position signals were low-pass filtered at 5 Hz (cut-off frequency, moving average finite impulse response filter, 17 filter taps, zero-phase) before velocities were extracted by computing first order, finite differences. Thereafter, the brain and stimuli signals were merged and resampled at 10 Hz. Then, the previously marked trials were rejected. Optionally, samples at various lags were concatenated to extend the feature space before fitting a regression model.

### Eye artifact correction

The eye artifact correction approach is based on a block design^19,42^. We fitted a linear eye artifact model to the recordings of blocks 2 and 4 (eye artifacts and resting brain activity) and applied the correction to the data of block 3.

The eye artifact model assumes a linear and stationary mixing of eye artifact sources ${{\bf{s}}}^{({\rm{a}})}({\rm{t}})$ (${n}_{artifactsources}\times 1$) with brain activity, denoted as noise ${\bf{n}}({\rm{t}})$ (${n}_{artifactsources}\times 1$). The activity at the EEG and EOG channels ${\bf{x}}(t)$ (${n}_{channels}\times 1$) is then

2 $${\bf{x}}({\rm{t}})={{\bf{A}}}^{(a)}{{\bf{s}}}^{({\rm{a}})}({\rm{t}})+{\bf{n}}({\rm{t}})$$

with a ${n}_{channels}\times {n}_{artifactsources}$ mixing matrix ${{\bf{A}}}^{({\bf{a}})}$. The brain activity ${{\bf{x}}}_{c}(t)$ can be recovered by subtracting the eye artifact activity at each channel

3 $${{\bf{x}}}_{{\rm{c}}}({\rm{t}})={\bf{x}}({\rm{t}})-{\hat{{\bf{A}}}}^{({\rm{a}})}{\hat{{\bf{s}}}}^{({\rm{a}})}({\rm{t}})\approx {\bf{n}}({\rm{t}})$$

if ${\hat{{\bf{A}}}}^{({\bf{a}})}$ and ${\hat{{\bf{s}}}}^{({\rm{a}})}(t)$ are good estimates of the unknown true mixing matrix and eye artifact sources. We applied the artifact subspace subtraction algorithm^19,43^ to compute the estimates. The algorithm estimates the source signals ${\hat{{\bf{s}}}}^{({\rm{a}})}(t)$ by linearly combining all channels

4 $${\hat{{\bf{s}}}}^{({\rm{a}})}({\rm{t}})={{\bf{V}}}^{({\rm{a}})}{\bf{x}}({\rm{t}})$$

with a ${n}_{artifactsources}\times {n}_{channels}$ unmixing matrix ${{\bf{V}}}^{({\rm{a}})}$. The correction in Equation (3) simplifies to

5 $${{\bf{x}}}_{{\rm{c}}}({\rm{t}})={\bf{x}}({\rm{t}})-{\hat{{\bf{A}}}}^{({\rm{a}})}{\hat{{\bf{s}}}}^{({\rm{a}})}({\rm{t}})=({\bf{I}}-{\hat{{\bf{A}}}}^{({\rm{a}})}{{\bf{V}}}^{({\rm{a}})}){\bf{x}}({\rm{t}})$$

The eye artifact model parameters (${\hat{{\bf{A}}}}^{({\rm{a}})}$ and ${{\bf{V}}}^{({\rm{a}})}$) were estimated in a two step approach^19^. First, penalized logistic regression was used to estimate each eye artifact source signal (e.g. horizontal eye movements) and its associated mixing coefficients (columns of ${\hat{{\bf{A}}}}^{({\rm{a}})}$). Second, given the mixing matrix ${\hat{{\bf{A}}}}^{({\bf{a}})}$ and the covariance matrix of the channels during resting brain activity ${{\bf{R}}}_{n}$ (${n}_{channels}\times {n}_{channels}$), the unmixing matrix ${{\bf{V}}}^{({\rm{a}})}$ can be computed via regularized weighted least squares^43^:

6 $${{\bf{V}}}^{({\rm{a}})}={({\hat{{\bf{A}}}}^{({\rm{a}}){\rm{T}}}{{\bf{R}}}_{n}{\hat{{\bf{A}}}}^{({\rm{a}})}+{\boldsymbol{\Lambda }})}^{-1}{\hat{{\bf{A}}}}^{({\rm{a}}){\rm{T}}}{{\bf{R}}}_{n}$$

with ${\boldsymbol{\Lambda }}$ being a ${n}_{artifactsources}\times {n}_{artifactsources}$ diagonal regularization matrix. The original publication^19^ contains details about the model fitting procedure, choice of regularization parameters and a comparison to state of the art eye artifact correction approaches.

### Movement parameter estimation

As low-frequency EEG is strongly correlated over time and space, there is considerable multicollinearity among the extracted features. An application of the partial least squares (PLS) regression^44^ method is particularly suitable in this scenario. As in^22^, we fit one model per movement parameter, condition and participant.

Let ${\bf{X}}$ be a $F\times N\,\,$matrix of $F$ predictor variables with $N$ samples (i.e, the EEG data), and let ${\bf{y}}$ be a $1\times N$ vector representing the dependent variable (i.e, a particular movement parameter). The predictor variables are modelled as

7 $${\bf{X}}={\bf{P}}{\bf{T}}+{\bf{E}}$$

with ${\bf{T}}$ representing a $D\times N$ matrix of latent components and ${\bf{E}}$, a $F\times N$ matrix of additive independent and identically distributed (iid) noise. ${\bf{P}}$, representing a $F\times D\,\,$matrix, projects the latent components ${\bf{T}}$ to the observed predictors ${\bf{X}}$. The goal of applying PLS regression is to find latent components ${\bf{T}}$ that have maximal covariance with the dependent variable ${\bf{y}}$, while reducing the dimension from $F$ to $D$. The dependent variable is then modelled as

8 $${\bf{y}}={{\bf{v}}}^{{\rm{T}}}{\bf{T}}+{\bf{g}}$$

with ${\bf{v}}$, representing a $D\times 1$ weight vector, and additive iid noise ${\bf{g}}$. Here, we applied the SIMPLS algorithm^23^ to estimate ${\bf{P}}$ and ${\bf{v}}$ for $D\,=\,10$ latent components. The estimates can be combined to a $F\times 1$ weight vector to directly estimate the dependent variable

9 $$\hat{{\bf{y}}}={\hat{{\bf{w}}}}^{{\rm{T}}}{\bf{X}}$$

from the predictor variables ${\bf{X}}$.

The model was evaluated by applying 10 times a 5-fold cross validation (CV). That is, the data was randomly partitioned to 5 folds. Then model parameters were fit to 4 folds. Model prediction was tested on the held out fold by computing the Pearson correlation coefficient ${r}_{y\hat{y}}$ between ${\bf{y}}$ and $\hat{{\bf{y}}}$. This was repeated until each fold was tested once. Thereafter, the random partitioning was repeated another 9 times, resulting in 50 estimates of ${r}_{y\hat{y}}$.

Chance level performance was estimated by applying the 5-fold CV to shuffled data. We broke the association between ${\bf{X}}$ and ${\bf{y}}$ while maintaining the correlation structure by randomly exchanging ${\bf{y}}$ across trials. The shuffling and 5-fold CV procedure was repeated 100 times.

To interpret the extracted models, we transformed weight vectors to activation patterns^25^. We scaled the unit-less patterns^45^ with the standard-deviation of $\hat{{\bf{y}}}$ to express the patterns in terms of voltages. The scaled pattern associated with an estimated weight vector is then

10 $$\hat{{\bf{a}}}={\hat{{\boldsymbol{\Sigma }}}}_{{\bf{X}}}\hat{{\bf{w}}}{\hat{\sigma }}_{\hat{{\bf{y}}}}^{-1}$$

with empirical covariance matrix ${\hat{{\boldsymbol{\Sigma }}}}_{{\bf{X}}}$ and standard-deviation ${\hat{{\rm{\sigma }}}}_{\hat{{\bf{y}}}}$ of predictors and estimated dependent variable, respectively. Analytical shrinkage regularization^46^ was applied to compute the estimate ${\hat{{\boldsymbol{\Sigma }}}}_{{\bf{X}}}$. We then summarized the 50 CV models by computing the geometric median^47^ across their patterns. This procedure yielded a representative pattern per movement parameter, condition and participant. To summarize the patterns obtained from chance level models, we randomly picked 50 patterns associated with 10 out of all 100 repetitions and computed their geometric median.

### Pattern source mapping

We applied EEG source imaging^26,27^ to map the scaled patterns from channel space (i.e, scalp level) to source space (i.e, cortical surface). Head models were created by co-registering the ICBM152 boundary element model (BEM) template^48^ with recorded electrode positions (ELPOS, Zebris Medical Gmbh, Germany) using the open source software Brainstorm^49^ version 19-Jan-2018. The BEM comprised three layers (cortex, skull, scalp) with relative conductivities (1, 0.008, 1). The cortex was modelled with 5001 voxels. BEM and electrode positions were co-registered by three anatomical landmarks (nasion, left and right preauricular points). Due to deviations between participant and template anatomy, we completed the co-registration by projecting floating electrodes to the scalp layer (Fig. S2b). OpenMEEG^50,51^ was used to compute the forward model; that is, to describe the propagation of the electric fields from cortex to scalp. sLORETA^52^ was applied to compute the corresponding inverse model for unconstrained sources. For unconstrained sources the activity at each voxel is described 3 components (x, y, z coordinates). We used three minutes of resting EEG, recorded during blocks two and four, to estimate sensor noise. The pre-processing of resting EEG was conducted indentically as explained above. We then estimated the noise covariance matrix by applying analytical shrinkage regularization^46^.

Before mapping the regression model patterns to source space, we normalized them to alleviate participant-dependent scaling. Since the scaled patterns reflected the potential at the scalp, their magnitude reflected the magnitude of the recorded signals. However, in the EEG, the global field power can vary considerably among participants. To compensate for this effect, we normalized the patterns by the average channel power. The average channel power was estimated by taking the median of the diagonal elements of the noise covariance matrix. The inverse scalar was then applied to scale participant-specific patterns. We then projected the final channel space patterns onto source space in Brainstorm, extracted the Euclidean norm of the three components (x, y, z coordinates) per voxel and optionally averaged over lags if the model comprised multiple lags.

### Source space statistics

Group level analysis was performed by computing paired differences between patterns of a movement parameter in source space. Significance was assessed at eight regions of interest (ROI)s, which have consistently been associated with movement processing. The ROIs are depicted in Figure 4c and cover fronto-central, primary sensorimotor, parietal and parieto-occipital areas. Activity at each ROI was summarized by the mean of its voxels. Significant ROIs were detected by applying two-tailed non-parametric permutation paired *t*-tests^53,54^ with 1000 repetitions. Regarding multiple comparisons, we controlled the false discovery rate (FDR) at a significance level of 0.05 by adjusting the *p*-values^55^.

### Code availability

The codes used for data collection and analysis in this study are available from the corresponding author upon request.

## Electronic supplementary material

Supplementary Video

Supplementary Material

## Data Availability

The data that support the findings of this study are available from the corresponding author upon request.
