# Supplementary material for: Tuning characteristics of low-frequency EEG to positions and velocities in visuomotor and oculomotor tracking tasks
Source: Sci Rep. 2018 Dec 7;8:17713. doi: 10.1038/s41598-018-36326-y (PMC6286357; doi:10.1038/s41598-018-36326-y)
Supplement: Supplementary file 2 — Supplementary Material [file 41598_2018_36326_MOESM2_ESM.pdf]

Supplementary Materials

Tuning characteristics of low-frequency EEG to positions and velocities in visuomotor and oculomotor tracking tasks

Reinmar J. Kobler[1], Andreea I. Sburlea[1], Gernot R. Müller-Putz[1*]

[1]Institute of Neural Engineering, Graz University of Technology, Austria
[*]e-mail: gernot.mueller@tugraz.at

Supplementary methods

Cursor trajectory replay procedure. In observation condition trials, we replayed the most recent matching executed cursor trajectory. Two cursor trajectories were considered as matching if they were associated with the same base target trajectory.

Before the experiments were conducted, we generated a pool of 12 base target trajectories from pink noise. We extended the pool to a total of 96 target trajectories by rotating (90°, 180° and 270°) and mirroring the base target trajectories. We distributed 90 of the target trajectories randomly over the 180 trials (once per condition). For each trial, we saved the associated base target trajectory and its angle of rotation and if it was mirrored. Online, the trial's target trajectory determined the target positions during the tracking period.

At the beginning of each experiment we used the first block (familiarization) to collect one participant specific executed cursor trajectory for each base target trajectory. These cursor trajectories were used as base cursor trajectories in the main experiment (third block). For each observation condition trial, we selected online the base cursor trajectory and rotated and mirrored it in a similar fashion as the base target trajectory for the particular trial. During the course of the experiment, we adaptively updated the base cursor trajectories. That is, after an execution condition trial was completed, the cursor trajectory of this trial was transformed back (rotation, mirroring) to the space of the base target (and cursor) trajectory. The new base cursor trajectory replaced the previous one.

The adaptive approach ensured that we could randomly distribute the target trajectories and conditions across the trials and, therefore, reduce any familiarization effects. The approach also ensured that the tracking dynamics between execution and observation condition did not differ.

Supplementary figures and tables

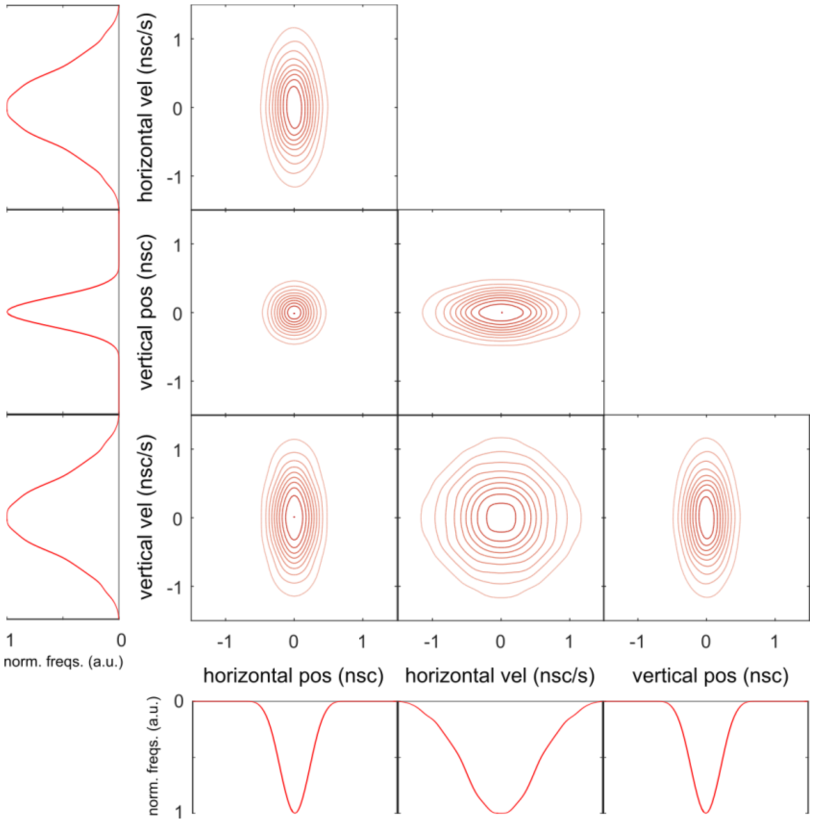

Figure S1: Marginal and bivariate distribution of target trajectory position and velocity signals. Positions and velocities are plotted in normalized screen coordinates (nsc) and nsc per second, respectively. Marginal distributions were estimated with a Gaussian density kernel and normalized by the maximal frequency. Bivariate distributions were estimated by computing a 2D histogram for a grid, spanning 50x50 points and subsequent convolution with a 2D Gaussian kernel.

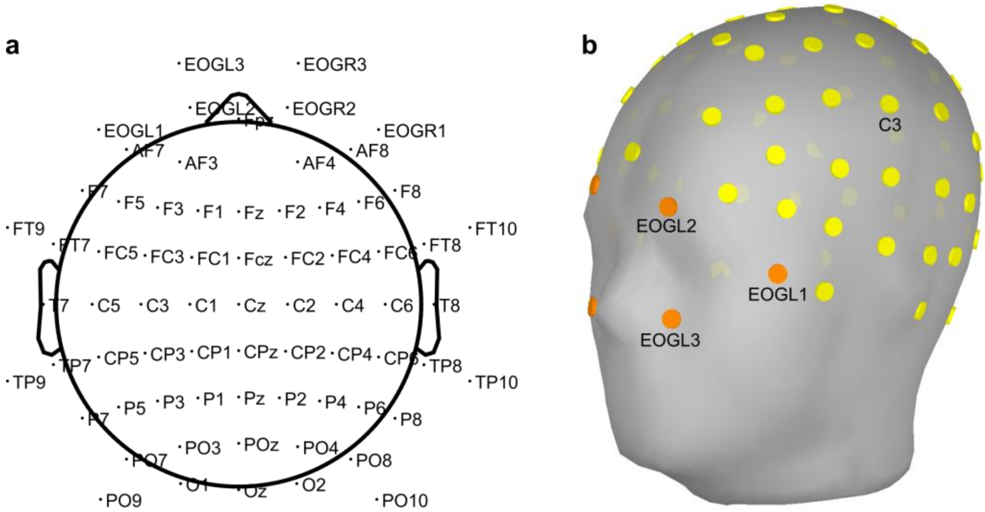

Figure S2: High density EEG and EOG electrode locations. **a,** Topographical representation of EEG and EOG electrode locations. **b,** Grand average electrode locations on the template head model after co-registration with the recorded electrode positions.

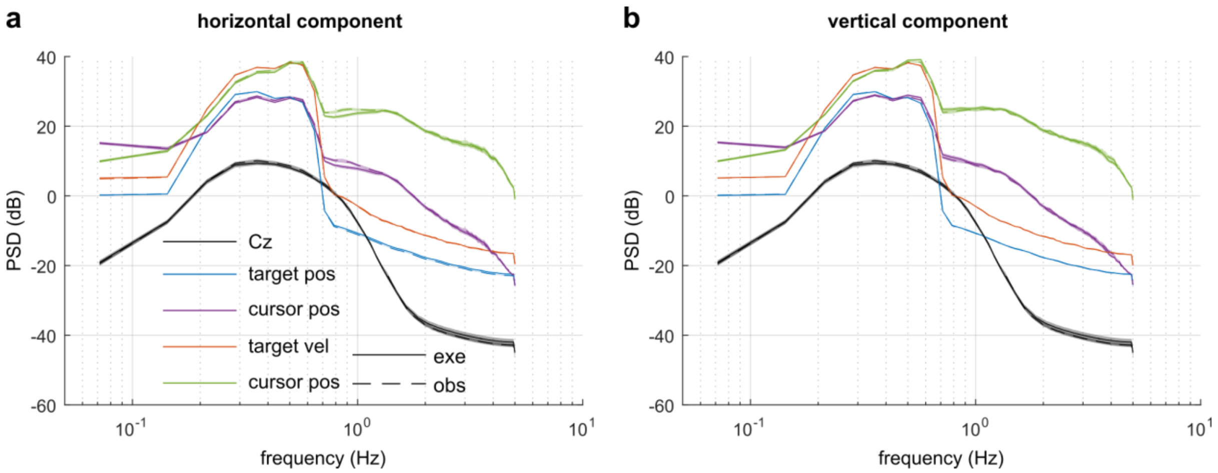

Figure S3: Grand average power spectral densities (PSD) of the movement parameter signals and the preprocessed EEG with Cz as a representative. **a,** PSDs in the execution (solid lines) and observation (dashed lines) conditions for the horizontal component. Shaded areas represent the standard-error of the mean. **b,** As in **a** for the vertical component.

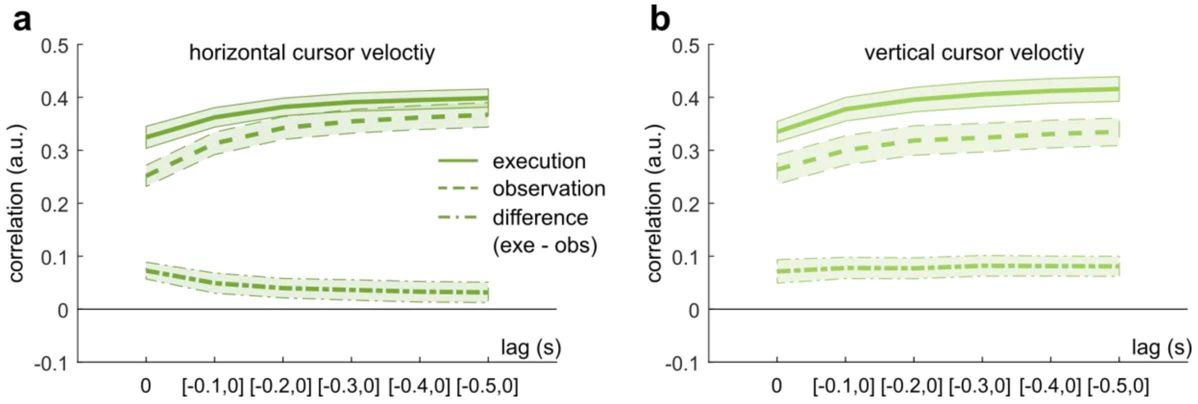

Figure S4: Grand average correlations between recorded and decoded cursor velocities for increasing prediction window sizes. **a,** Results for the horizontal component. **b,** As in **a** for the vertical component.

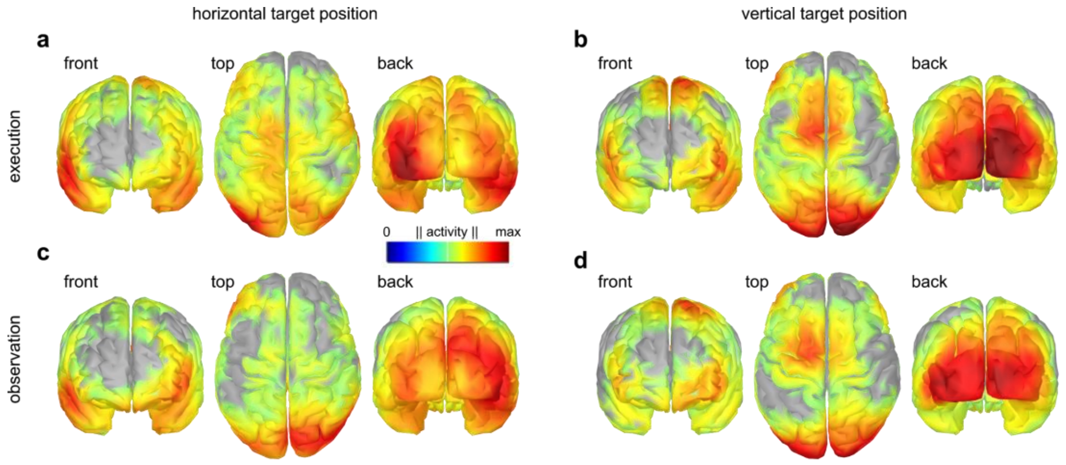

Figure S5: Grand average single-lag (lag 0) pattern activity for target position decoders. **a,b,** Patterns in the execution condition for horizontal **a** and vertical **b** components. **c,d,** As in **a,b** for the observation condition. Pattern activity indicates that the decoders relied on a mixture of brain activity (occipital, parietal and fronto-central) and eye artifacts in both conditions. Since the solution space was limited to the cortical surface, eye artifact activity was reflected on rostral areas in the frontal and temporal lobes.

Table S1: Data cleaning summary per participant. Column two indicates the interpolated EEG channels. The other columns list the number of trials marked for rejection by the application of three criteria. Column three summarizes the EEG signal outliers (larger than $\pm$200µV or abnormal probability or kurtosis); column four lists the number of trials with abnormal eye movement target correlation; and column five indicates how often the palm-position signal was lost. The last column summarizes the total number of trials rejected by combining all three criteria.

Participant	interpolated channels	EEG	target EOG corr.	tracking error	**total**
1	T7, T8, TP9	10	12	7	**26 (14%)**
2	T8, FT10, AF8	25	11	3	**37 (21%)**
3	FT10	5	11	0	**15 (8%)**
4	F5	11	8	7	**22 (12%)**
5	TP10, PO3	15	11	4	**24 (13%)**
6	TP9	21	14	3	**38 (21%)**
7	-	11	8	5	**22 (12%)**
8	TP7, TP8, TP9, TP10	30	8	3	**41 (22%)**
9	T7	14	5	3	**21 (12%)**
10	F7, AF8, T8, PO10	23	6	19	**45 (25%)**
11	TP10	18	18	2	**34 (19%)**
12	FC5, T8	14	3	9	**24 (13%)**
13	T8	21	12	5	**35 (19%)**
14	T7, T8, TP7, TP9, TP10	17	14	2	**31 (17%)**
15	FC3, TF7	5	3	2	**10 (6%)**
**average**	**2.1**	**16 (9%)**	**9 (5%)**	**5 (3%)**	**28 (16%)**
